# How to treat cardiovascular autonomic failure in Parkinson’s disease

**DOI:** 10.1007/s00702-025-03096-7

**Published:** 2026-01-03

**Authors:** Alessandra Fanciulli, Fabian Leys, Günter Höglinger, Wolfgang H. Jost

**Affiliations:** 1 Department of Neurology, Medical University of Innsbruck, Innsbruck, Austria; 2 Department of Neurology, LMU University Hospital, Ludwig-Maximilians-Universität (LMU) München, Munich, Germany; 3 Deutsches Zentrum für Neurodegenerative Erkrankungen (DZNE), Munich, Germany; 4 Munich Cluster for Systems Neurology (SyNergy), Munich, Germany; 5 Parkinson-Klinik Ortenau, Wolfach, Germany

**Keywords:** Parkinson’s disease, Orthostatic hypotension, Post-prandial hypotension, Supine hypertension, Nocturnal hypertension

## Abstract

Cardiovascular autonomic failure is a frequent non-motor feature of Parkinson’s disease (PD) that affects up to one third of individuals from the premotor to the advanced stages of the disease, with major diagnostic, therapeutic and prognostic implications. It may manifest with orthostatic, post-prandial or exercise-induced hypotension, as well as hypertensive episodes in the supine position during wakefulness or nocturnal sleep. Hypotensive episodes may remain asymptomatic or manifest with symptoms of end-organ hypoperfusion in the upright position, after meals or during exertion that may include lightheadedness, blurred vision, cognitive slowness, shuffling gait, back pain, fatigue or, in severe cases, syncope. Supine and nocturnal hypertension are likewise often asymptomatic, yet may cause nocturnal polyuria, and disrupt sleep through frequent nocturnal toilet visits. Bedside screening for cardiovascular autonomic failure relies on targeted history taking, eventually supported by validated questionnaires, and supine to standing heart rate and blood pressure measurements. A more detailed assessment is obtained with cardiovascular autonomic function tests under continuous, non-invasive, hemodynamic monitoring, complemented by 24-hours ambulatory blood pressure monitoring and home blood pressure diaries. Hypotensive episodes are managed by addressing potential triggers, such as infections, anemia, dehydration and polypharmacy, followed by a stepwise implementation of behavioral, non-pharmacological and pharmacological strategies. Individuals with orthostatic hypotension should be constantly monitored for concomitant supine and nocturnal hypertension, especially if treatment with pressor agents has been recently started. Hypertensive episodes are likewise treated in a stepwise fashion with preventive, non-pharmacological and pharmacological measures, prioritizing hypotension control during daytime and mitigating hypertension overnight.

## Introduction

Cardiovascular autonomic failure is a frequent non-motor symptom of Parkinson’s disease (PD) (Barone et al. 2009) and is due to neurodegenerative changes primarily affecting the efferent branch of the arterial baroreflex arch (Kaufmann et al. 2020). Its main clinical manifestation is orthostatic hypotension (OH), classically defined by a sustained fall in systolic blood pressure (BP) ≥ 20 mmHg and/or diastolic ≥ 10 mmHg within three minutes in the upright position (Freeman et al. 2011). Individuals with OH may experience orthostatic syncope or, more frequently, unexplained falls (Finucane et al. 2017), lightheadedness, cognitive slowing, impaired vision, fatigue, shortness of breath and pain in the neck and shoulders (i.e., coat-hanger pain), or in the lower back. These symptoms are nonspecific and may also occur in a variety of non-neurological conditions (Fanciulli et al., 2017), but in OH, they typically develop upon standing and resolve when sitting or lying down (Freeman 2008). In some cases, OH may remain asymptomatic, largely depending on individual cerebrovascular autoregulatory mechanisms (Indelicato et al. 2015) and whether the BP falls in the hypotensive range upon standing (Palma et al. 2015).

On average, OH affects every third person living with PD (Velseboer et al. 2011). It may already emerge in the premotor stages of the disease as an isolated, often under recognized, autonomic presentation of PD, with important implications for biomarker discovery, the design of disease-modifying trials and, more broadly, for our understanding of the mechanisms driving α-synuclein pathological spreading (Giannini et al. 2018; Coon et al. 2020; Horsager et al. 2020; Fanciulli and Wenning 2021; Millar Vernetti et al. 2024; Andersen et al. 2025). In older patients or those with more advanced disease, the prevalence of OH may further increase due to non-neurogenic contributors, including cardiovascular comorbidities, dehydration, polypharmacy and age-related changes in the cardiovascular and autonomic nervous systems (Ricci et al. 2015; Rivasi et al. 2020).

**Fig. 1:**
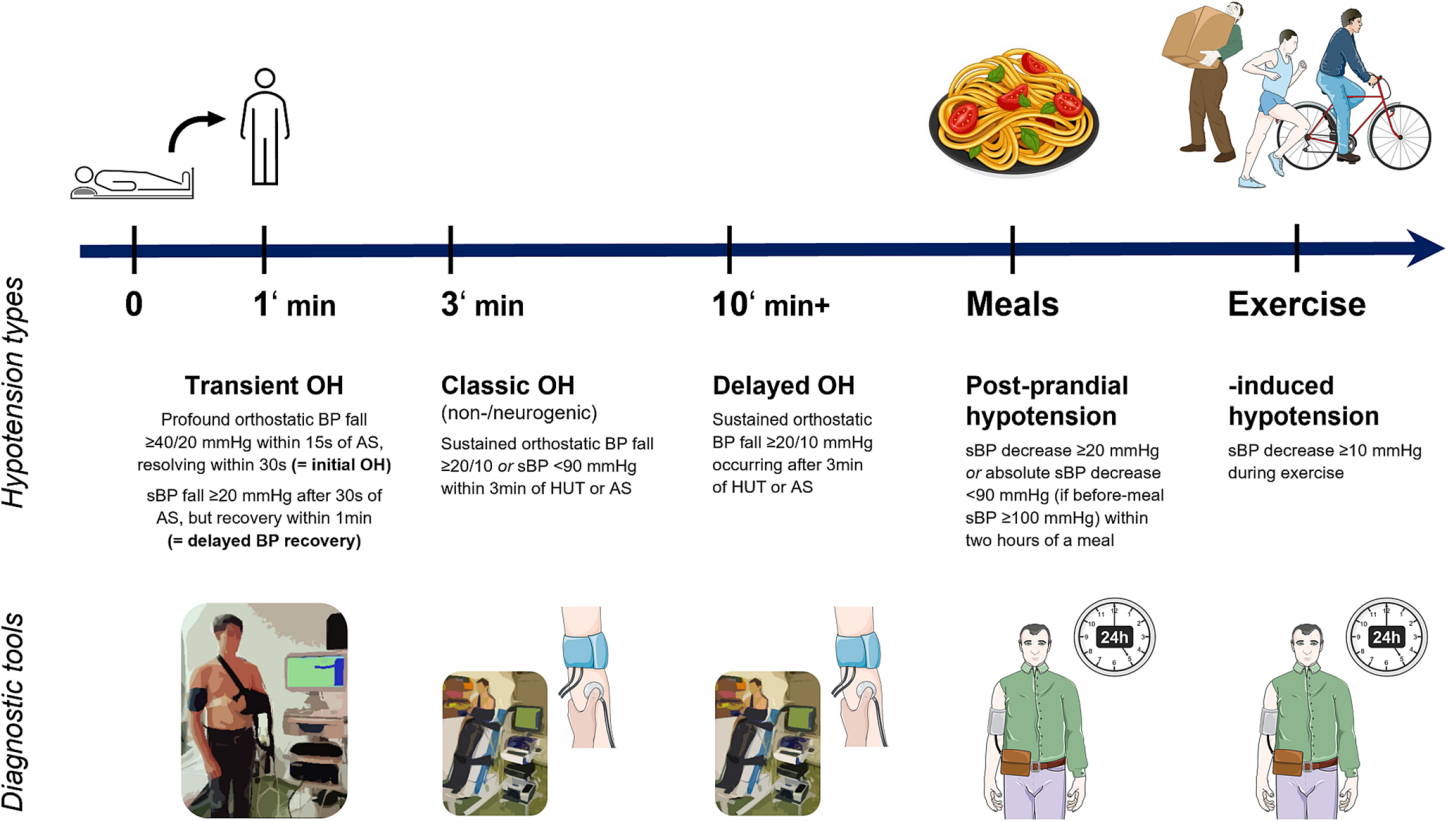
Types of hypotension in Parkinson’s disease. *AS* active standing, *HUT* head-up tilt, *sBP* systolic blood pressure

Beyond classic OH, cardiovascular autonomic failure may also manifest with transient, severe BP falls immediately upon standing, referred to as transient OH, which may be as common as classic OH in PD and further increase the risk of orthostatic intolerance and syncope-related falls (Fanciulli et al. 2020a). In some cases, OH may only manifest after prolonged standing, a condition known as delayed OH (Calio et al. 2025). In addition, vasodilation in the splanchnic district after meals or in active muscle groups during exercise may trigger BP falls, which are insufficiently compensated in the setting of baroreflex failure, leading to post-prandial and exercise-induced hypotension in a substantial proportion of individuals with PD (Low et al. 2014; Pavelic et al. 2017). An overview of the types of hypotension that may develop in individuals with PD is reported in Fig. 1, along with their definitions.

Every second person with PD and OH may also develop supine hypertension (SH), which may disrupt the circadian BP rhythm, and, in severe cases, cause nocturnal hypertension (Fanciulli et al. 2014, 2016a, 2018). The mechanisms leading to SH in PD are not fully understood, but likely involve baroreflex dysfunction, sensitization of vascular adrenoreceptors following autonomic denervation, individual predisposition, and other neuro-humoral mechanisms including aberrant vasopressin and renin-angiotensin release (Shannon et al. 2000). Medications for treating OH may exacerbate SH and nocturnal hypertension, underscoring the need to address both hypotensive and hypertensive manifestations of cardiovascular autonomic failure in clinical practice. This article provides a practical framework for screening, diagnosing, and treating OH and post-prandial hypotension in PD, with particular attention to their interaction with supine and nocturnal hypertension.

## Diagnostic work-up

In clinical practice, the first and most important step for identifying cardiovascular autonomic failure in PD is an active surveillance for relevant symptoms. Many affected individuals may not recognize these symptoms as cardio-circulatory in origin or associate them with PD, and therefore tend to underreport them. Careful questioning about falls unrelated to slips or trips, particularly if occurring after meals, heat exposure, alcohol intake or physical exercise, or about any symptoms, including back pain, that worsen upon standing or after dopaminergic medication intake, can be highly informative. Several structured questionnaires are available for screening autonomic symptoms. Some cover the full spectrum of PD non-motor symptoms, like the Non-motor Symptoms Scale (Chaudhuri et al. 2007) and the Part I of the MDS-Unified Parkinson Disease Rating Scale (Goetz et al. 2008), while others, including the SCOPA-AUT Scale for Outcomes in Parkinson‘s disease and the Orthostatic Hypotension Questionnaire (OHQ), specifically focus on autonomic and OH-related symptoms (Visser et al. 2004; Kaufmann et al. 2012).

A standing or *Schellong test*, named after Fritz Schellong, a German cardiologist who pioneered research on baroreflex physiology, consists of measuring heart rate and BP changes during the transition from the supine to the upright position using oscillometric arm-cuff measurements (Brignole et al. 2018; Fanciulli et al. 2019a). It is quick and widely available, therefore representing an excellent tool for bedside and outpatient OH and SH screening, especially in countries or regions with no autonomic referral centers (Habek et al. 2022). Oscillometric arm-cuff or wrist BP devices are, however, prone to errors, particularly in the presence of arrhythmias, tremor, infrequent calibration or incorrect placement (Teuschl et al. 2025). For a more accurate cardiovascular autonomic assessment, laboratory-based, beat-to-beat, non-invasive hemodynamic monitoring during orthostatic and other baroreflex challenges, like the Valsalva maneuver, is preferable (Fanciulli et al. 2014; Cheshire et al. 2021). These tests help to differentiate neurogenic OH, caused by neurodegenerative changes of the autonomic nervous system and marked by an inadequate heart rate response to orthostatic BP falls, from non-neurogenic causes of BP instability like hypovolemia or polypharmacy, where the orthostatic heart rate response and hemodynamic counter-regulation during the Valsalva maneouver are preserved (Norcliffe-Kaufmann et al. 2018). Continuous hemodynamic monitoring during active standing can also detect forms of OH missed by first-line bedside tests, particularly transient OH, too short-lived for being captured by oscillometric measurements (Wieling et al. 2007; Benditt et al. 2025), and delayed OH, which requires a prolonged orthostatic challenge, often unfeasible or uncomfortable in outpatient settings (Calio et al. 2025).

Twenty-four-hour ambulatory BP monitoring (ABPM) further helps to detect other autonomic features, including postprandial and exercise-induced hypotension (Leys and Fanciulli 2022), as well as supine and nocturnal hypertension (Sommer et al. 2011; Fanciulli et al. 2018). These often remain asymptomatic, apart from unspecific malaise, headache or frequent nocturnal toilet visits due pressure natriuresis overnight, and therefore require an active screening. To improve the diagnostic yield of ABPM, patients should be encouraged to engage in activities that might have triggered symptoms, like walking or biking, and to compile a detailed diary noting meal times, activities, sleep and medication intake.

Home BP diaries are another valuable and affordable tool, offering insights into BP levels in daily life and outside of medical settings. To ensure reliability, patients should be carefully instructed on the correct device positioning, and asked to record not only seated, but also supine and standing BP values, ideally over two to three consecutive days and at different times (i.e., in the morning, after lunch and at bedtime) (Fanciulli et al. 2020b). This may help to capture both day to day and diurnal fluctuations in OH and SH severity. Given their low cost and replicability, home BP measurements are particularly useful for monitoring the efficacy and eventual side effects of implemented interventions.

## Treatment of orthostatic hypotension

### Addressing non-neurogenic causes and aggravating factors

In all individuals newly diagnosed with OH, it is essential to explain them the hemodynamic nature of their disturbances, the likelihood of recurrence and strategies to reduce the risk of injurious falls and syncope (Brignole et al. 2018). If the clinical assessment reveals aggravating factors, such as dehydration, severe anemia or any infectious state, these should be treated first. The medication schedule should be also reviewed for drugs with hypotensive effects, including centrally acting drugs such as opioids, hypnotics, anti-depressants (trazodone, in particular), gabapentinoids and neuroleptics, which might need to be tapered or discontinued (Rivasi et al. 2020). Especially when the onset of symptomatic OH coincides with the initiation or dose escalation of such drugs, a careful risk-benefit assessment is recommended.

In PD practice, OH may sometimes worsen during the titration of dopaminergic agents, likely due to their effect on renal water and salt excretion (Harris and Zhang 2012; Jost et al. 2020). Several studies reported a dose-dependent hypotensive effect following both L-Dopa and dopamine agonists administration (Kujawa et al. 2000; Nimmons et al. 2022; Su et al. 2023; Cani et al. 2024; Earl et al. 2024; Longardner et al. 2025). While direct comparisons among dopaminergic agents remain limited and sometimes hampered by imprecise definitions of OH and OH-related symptoms, L-Dopa has been generally associated with lower risk of OH and other vasomotor adverse events like ankle edema compared with dopamine agonists (Nimmons et al. 2022; Crispo et al. 2024). Within the dopamine agonist class, studies indicated that ropinirole may be associated with a higher frequency of OH than pramipexole (Etminan et al. 2003), whereas rotigotine may confer lower odds of developing OH (Crispo et al. 2024). This latter observation may be due to its transdermal, continuous delivery, preventing excessive peaks in the plasmatic drug concentration. It remains uncertain whether switching from one dopamine agonist to another, or from a dopamine agonist to L-dopa, is in itself sufficient to control orthostatic symptoms in individuals with PD, who develop OH after initiation of dopaminergic therapy. Nevertheless, such a strategy may be considered on an individual basis, taking into account the motor demand, accompanying non-motor symptoms, and relevant comorbidities. While some patients may experience spontaneous improvement at follow-up or benefit from on-demand domperidone intake (Schoffer et al. 2007), others require OH-targeted interventions to maintain adequately dosed dopaminergic regimens and ensure good motor control.

Regarding device-aided PD therapies, a recent systematic review pooled findings of 40 studies examining the effects of deep brain stimulation (DBS) of the subthalamic nucleus on cardiovascular autonomic function (Cucinotta et al. 2024). Despite methodological heterogeneities concerning symptom assessments, inconsistent use of control groups, variable follow-up intervals and effect sizes, an improvement in orthostatic symptoms was commonly reported. According to the available evidence, such symptomatic improvement may be largely attributable to post-operative reductions in L-Dopa equivalent daily dose. Some studies incorporating objective cardiovascular autonomic assessments reported enhanced baroreflex sensitivity and skin sympathetic responses with stimulation ON versus OFF, possibly indicating direct effects of DBS on central autonomic outflow (Cucinotta et al. 2024). Pooled analyses of trials on L-Dopa/Carbidopa intestinal gel infusions have also highlighted beneficial effects on OH, likely due to improved mobility, fluid intake and stabilization of L-Dopa plasmatic fluctuations (Galli et al. 2025).

### Non-pharmacological measures

**Fig. 2:**
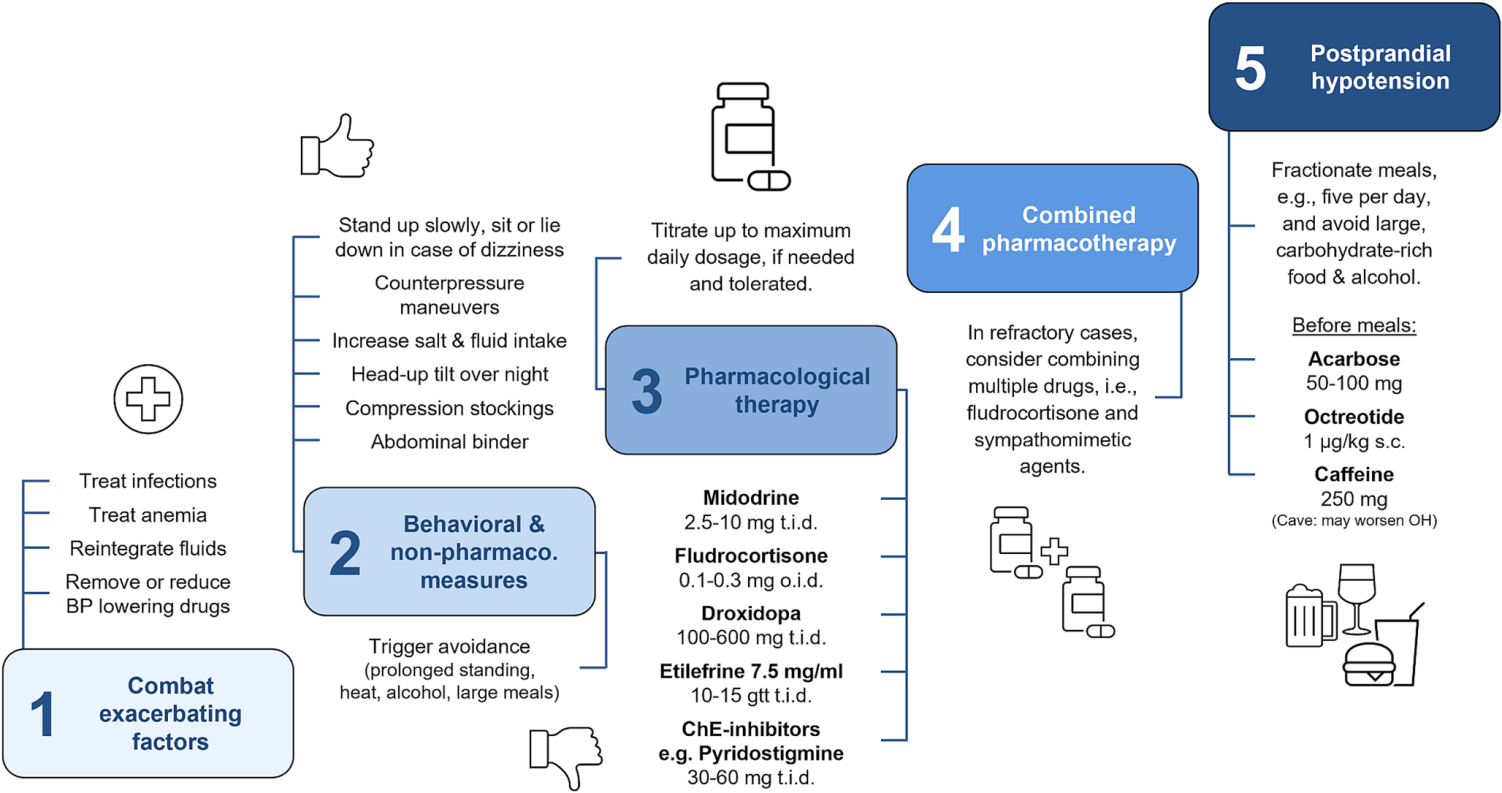
Stepwise treatment of orthostatic and post-prandial hypotension in Parkinson’s disease

When no modifiable cause of OH can be identified, or when symptoms persist despite addressing potentially exacerbating factors, a stepwise therapeutic approach should be implemented tailored to the symptoms severity and entailing behavioral, non-pharmacological, and pharmacological strategies [see Fig. 2, Gibbons et al. (2017)].

Behavioral measures to reduce OH burden include slow changes from the supine to the upright position, including pausing in the seated position to prevent abrupt BP falls. Typical OH triggers, such as large meals, alcohol intake, heat exposure, dehydration and prolonged standing, should be carefully avoided (Gibbons et al. 2017). Simple adaptations like showering or, for male individuals, emptying the bladder in a seated position may reduce the risk of syncope during daily routines (Fanciulli et al. 2020b).

Non-pharmacological strategies to combat OH include head-up tilted sleeping by 10° or more, which promotes overnight vasopressine release and reduced pressure natriuresis (Ten Harkel et al. 1992), increasing daily fluid intake up to 2.5 L, especially in the first part of the day, and dietary salt (6–10 g) (Eschlbock et al. 2017). These interventions should be, however, applied with caution in individuals with concomitant cardiac, renal or hepatic conditions.

Isometric maneuvers like stepping on the place, tiptoeing, crossing the legs, clenching the fists, or tensing abdominal and gluteal muscles effectively counteract the BP upon standing and can be performed in case of dizziness, when sitting or lying down is not immediately possible (Wieling et al. 2015). Physical exercise, particularly water training and exercises performed in the seated or supine position may be also considered to improve orthostatic tolerance (Bane et al. 2024; Dunlap et al. 2025).

The use of abdominal binders may further reduce splanchnic venous pooling and improve OH (Fanciulli et al. 2016b; Okamoto et al. 2016). Compression stockings alone are generally insufficient to combat OH and may be difficult to wear (Newton and Frith 2018), but can be considered as an adjunct, especially if ankle edemas are present.

### Pharmacological measures

If non-pharmacological interventions fail to adequately control OH symptoms, pharmacological treatments should be introduced. These may either enhance the vascular tone using sympathomimetic agents, or increase the vascular load by increasing the plasmatic volume (see Fig. 2).

The choice of pressor agents should be guided by OH severity, eventual comorbidities, and potential side effects. In early PD stages or in individuals with good motor performance, an intensified pharmacological regimen may be warranted to secure adequate OH control and reduce the risk of syncope. Conversely, in advanced, wheel-chair bound individuals, avoiding pharmacological interactions and reducing the risk of adverse effects may gain priority.

Among sympathomimetic agents, midodrine, a direct α1-adrenoceptor agonist, has been extensively studied and applied over the last decades in clinical practice to treat OH (Jankovic et al. 1993; Low et al. 1997; Wright et al. 1998; Kaufmann et al. 2002). Droxidopa, a noradrenaline precursor metabolized to noradrenaline by dopa-decarboxylase (Freeman et al. 1999; Kaufmann et al. 2003, 2014; Hauser et al. 2015, 2018), may be considered as an alternative, but it is not licensed in Europe. Other sympathomimetic agents, such as etilefrine, which has a similar pharmacodynamics profile to midodrine but was less investigated in PD, may be considered in case of supply chain disruptions or reimbursement issues. All abovementioned agents should be introduced at the lowest dose, administered three times a day, and gradually titrated based on symptoms severity. Atomoxetine, a noradrenaline reuptake inhibitor, has been postulated to raise the BP by prolonging the permanence of endogenous cathecolamines in the synaptic cleft. Although former evidence indicated a pressor effect in OH (Byun et al. 2020; Jung et al. 2023), more recent randomized controlled studies including individuals with PD failed to demonstrate a significant improvement in orthostatic symptoms (Mwesigwa et al. 2024). Similarly, evidence on pyridostigmine, a cholinesterase inhibitor that may indirectly stimulate post-ganglionic sympathetic transmission is mixed (Pavic et al. 2025). Most recent analyses indicate only mild effects, suggesting that pyridostigmine may be useful in case of milder OH or as adjunct therapy (Okamoto et al. 2025). Post-hoc analyses of trials in PD individuals with dementia treated with rivastigmine, another cholinesterase inhibitor, showed more favorable cognitive outcomes in subjects with concomitant OH, possibly through improved cerebral perfusion (Espay et al. 2021). All abovementioned direct and indirect sympathomimetic drugs should be used cautiously in individuals with concomitant cardiac disease, kidney failure, or urinary retention.

Fludrocortisone, a synthetic mineralocorticoid, increases plasma volume by promoting sodium retention and can be used alone or in combination with sympathomimetic drugs. Long-term comparisons of the efficacy and safety profile of single versus combined pressor agents are, however, lacking (Eschlbock et al. 2017). Fludrocortisone is contraindicated in individuals with heart or kidney failure, and electrolyte monitoring is required at treatment begin, as well as in case of fever or diarrhea.

Other agents such as yohimbine, ergotamine, dihydroergotamine, ephedrine, desmopressin, indomethacin, and fluoxetine have shown potential efficacy in case series and proof-of-concept studies. Many of these studies were however limited by short duration, heterogeneous study populations and uncertain safety profiles (Eschlbock et al. 2017). Such pharmacological options may be therefore considered in carefully selected cases with refractory OH, when other treatments proved ineffective or were not tolerated.

### Treatment of transient and delayed orthostatic hypotension

Patients with transient OH should be encouraged to regularly practice counterpressure maneuvers when standing up, as these may effectively contrast the initial orthostatic BP drop and associated symptoms. Additional behavioral strategies include avoiding prolonged sitting or lying, performing cognitive tasks such as mental arithmetics when standing-up, and using cooling garments (Krediet et al. 2007; Clarke et al. 2010; Sheikh et al. 2022). Deprescribing antihypertensive agents, particularly α1- and β-blockers, may also be beneficial in patients with transient OH. In refractory cases, pharmacological treatment with sympathomimetic agents can be considered (Lewis et al. 2013; Canney et al. 2016; Fanciulli et al. 2020a).

Recent studies showed that behavioral, non-pharmacological and pharmacological strategies to manage OH may be also effective for treating delayed OH (Calio et al. 2025).

## Targeted measures for post-prandial hypotension

Post-prandial hypotension may develop due to splanchnic vasodilation after any meal intake irrespective of the time of the day or the presence of OH, and whenever identified, targeted non-pharmacological and pharmacological strategies should be considered to mitigate the post-prandial BP fall and associated symptoms. Dietary modifications represent a key non-pharmacological approach. Reducing the amount of protein and carbohydrate intake per meal, either through dietary restrictions, substitution of dietary carbohydrates with low-nutritive or non-nutritive sweeteners, or the addition of guar gum, has been shown to attenuate post-prandial hypotension in affected individuals (Ong et al. 2014; Jang et al. 2015; Son and Lee 2015; Oberoi et al. 2022; Farrow et al. 2025). Similarly, rapid water ingestion before meals may help reduce the post-prandial BP fall (Shannon et al. 2002). Short walks after meals have also been demonstrated to mitigate post-prandial hypotension (Oberman et al. 1999; Nair et al. 2015).

Pharmacological adaptations are another important aspect of post-prandial hypotension management. Deprescribing antihypertensive agents, particularly α1-blockers and loop diuretics, should be evaluated in patients with post-prandial hypotension to minimize the magnitude of post-prandial BP reductions (van Kraaij et al. 1998; Schoevaerdts et al. 2019). In patients with post-prandial hypotension and non-insulin dependent diabetes mellitus, pharmacologic interventions targeting carbohydrate absorption may be beneficial. Acarbose and voglibose, for instance, have been reported to reduce post-prandial hypotension in this population (Maruta et al. 2006; Madden et al. 2015; Wang et al. 2021), and metformin may produce similar effects (Borg et al. 2019; Quast et al. 2025).

Other pharmacological approaches include caffeine intake up to 250 mg before meals, which may help to counteract the post-prandial BP fall (Onrot et al. 1985; Lipsitz et al. 1994). Higher caffeine doses should be, however, discouraged, as they may foster diuresis and result counterproductive (Seal et al. 2017). Droxidopa administration before meals may be also considered (Freeman et al. 1996). In patients who do not tolerate or respond to these strategies, subcutaneous octreotide may be considered as a therapeutic option to reduce both the post-prandial BP fall and associated symptoms under careful monitoring for side-effects like nausea, abdominal cramps and glycemic disturbances (Hoeldtke et al. 1986, 1989; Alam et al. 1995).

## Management of supine and nocturnal hypertension

The treatment of SH should be tailored to its severity, the presence of nocturnal hypertension and individual cardio- and cerebrovascular risk factors. The rationale of treating supine and nocturnal hypertension consists in reducing the risk of hypertensive emergencies, minimizing the overnight plasma volume loss, which may worsen OH the following day, and to prevent long-term organ damage (Fanciulli et al. 2013; Palma et al. 2020). These potential benefits must be carefully weighed against the risk of aggravating OH, thus increasing the risk of syncope and injurious falls. Valuable insights into the individual risk of hypertensive crises, on one hand, and syncope on the other, can be gained by the highest and lowest BP readings over the 24 h at ABPM (Fanciulli et al. 2018; Leys and Fanciulli 2022). A tight BP control, as recommended in the general ageing population, is usually not advisable (Jordan et al. 2019).

**Fig. 3:**
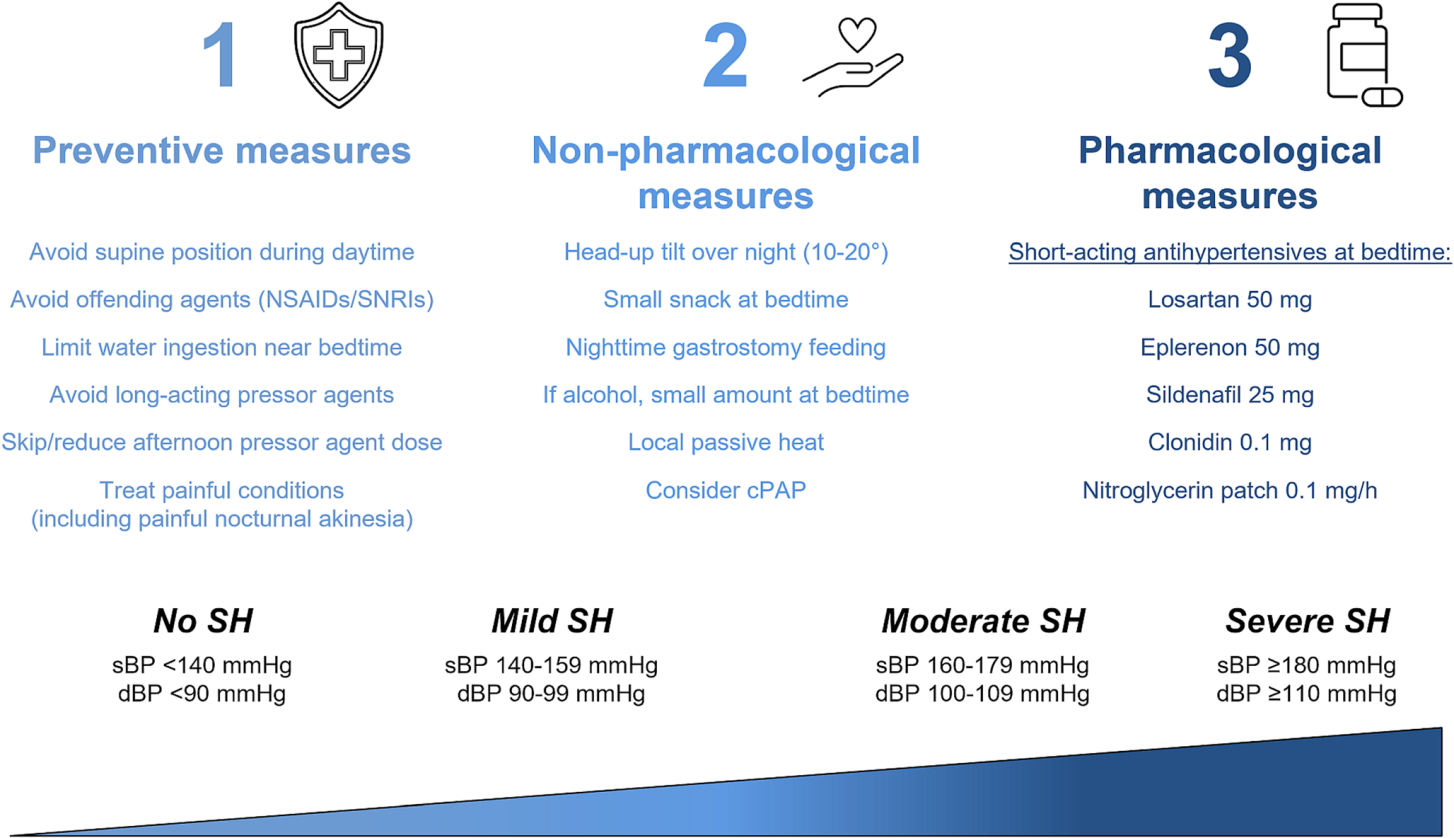
Management of supine and nocturnal hypertension in Parkinson’s disease. *cPAP* continuous positive airway pressure, *NSAIDs* non-steroidal anti-inflammatory drugs, *SNRIs* serotonin-norepinephrine reuptake inhibitors, *dBP* diastolic blood pressure, *sBP* systolic blood pressure, *SH* supine hypertension

In patients without SH, behavioral measures and therapeutic adaptations can reduce the risk of developing overt supine or nocturnal hypertension (see Fig. 3). Patients should avoid lying in the supine position during the day, especially when pressor agents are active. If daytime rest is desired, a semi-seated position is preferable. Short-acting pressor agents (e.g., sympathomimetic drugs) are generally preferred over long-acting agents (e.g., fludrocortisone), and the final dose should be scheduled no later than 4 p.m. If OH symptoms are less prominent in the second half of the day, the afternoon dose of the pressor agents may be tapered or even discontinued (Jordan et al. 2019). Drugs not primarily prescribed as pressor agents, but known to increase BP in patients with baroreflex dysfunction, such as NSAIDs, domperidone, or serotonin-norepinephrine reuptake inhibitors, should be avoided whenever possible. Similarly, water intake should be limited or restricted during the last part of the day, particularly near bedtime, to prevent any nocturnal pressor effect. Finally, painful conditions, including painful nocturnal akinesia due to wearing-off phenomena, should be adequately treated, as discomfort itself can contribute to nocturnal hypertension (Fanciulli et al. 2025).

In cases of mild to moderate supine or nocturnal hypertension, non-pharmacological measures should be introduced first (see Fig. 3). These include elevating the entire body at a 10–20° head-up tilt during sleep and consuming a small snack before bedtime to counteract SH with a post-prandial effect. Following the same principle, the main caloric intake may be scheduled in the evening hours for patients receiving enteral nutrition via gastrostomy (Young and Mathias 2008). Although alcohol is not recommended as a therapeutic measure, patients who consume small amounts may choose to do so before bedtime, as this may transiently lower BP. Additional strategies may include the use of localized passive heat overnight (e.g., warming blankets or warming bags) to induce vasodilation (Okamoto et al. 2021). Continuous positive airway pressure (cPAP) therapy has been shown to improve nocturnal hypertension through multiple putative mechanisms, likely entailing increased intrathoracic pressure with Valsalva-like effects (Okamoto et al. 2023), reduced apnea-driven nocturnal arousals, and mitigation of the vasoconstrictive effects of hypercapnia (Baker et al. 2025). A polysomnography and prompt treatment initiation should be therefore recommended to any individuals with PD and suspected sleep-disordered breathing at medical history (Fanciulli et al. 2025).

If the BP remains elevated overnight despite all abovementioned preventive and non-pharmacological measures, pharmacological therapy may be considered. Although the available evidence derives from short-term or single administration studies in small populations, several agents have been shown to reduce nocturnal hypertension in individuals with cardiovascular autonomic failure (see Fig. 3). These include eplerenone, losartan, sildenafil, clonidine, transdermal nitroglycerin, and nifedipine (Jordan et al. 1999; Shibao et al. 2006a, b; Gamboa et al. 2008; Arnold et al. 2013, 2016). Among these, losartan and clonidine also reduced nocturnal sodium excretion, which may have a favorable effect on OH severity the day after as well. By contrast, long-acting antihypertensives such as nifedipine were found to exacerbate OH symptoms the day after. Short-acting BP lowering agents should be therefore preferred for treating nocturnal hypertension in the context of autonomic failure and their administration should be restricted to the evening hours. During the daytime, the treatment priority should remain focused on effectively managing OH symptoms.

## Conclusion

Cardiovascular autonomic failure plays an important role in the differential diagnosis of PD, as it carries a high negative predictive value against tauopathies such as progressive supranuclear palsy and corticobasal syndrome, in which the autonomic nervous system remains largely spared (Wenning et al. 1998; Miki et al. 2019; Leys et al. 2022). By contrast, cardiovascular autonomic failure represent a core non-motor feature of other α-synucleinopathies like multiple system atrophy and dementia with Lewy bodies (Wenning et al. 2013; McKeith et al. 2017), and electrophysiological assessments provide overlapping findings (Leys et al. 2020). In these cases, it is the early development of multidomain autonomic failure, often combined with signs of rapid disease progression or cognitive impairment that points towards an alternative diagnosis to PD (Koga et al. 2015; Fanciulli et al. 2019b, 2024).

Among individuals with PD, cardiovascular autonomic failure has been more consistently associated with the postural instability and gait difficulty (PIGD) rather than with the tremor-dominant phenotype (Allcock et al. 2006; Marras and Chaudhuri 2016; Wang et al. 2020; Gu et al. 2023). In the context of the recently proposed brain-first versus body-first model of α-synuclein pathological propagation, involvement of peripheral and central cardiovascular autonomic relays is thought to preferentially characterize the body-first variant (Horsager et al. 2020; Andersen et al. 2025). Among genetic forms of PD, earlier and more severe cardiovascular autonomic failure has been described in pedigrees with SNCA gene mutations or triplications, as well in sporadic PD cases carrying SNCA single nucleotide polymorphisms, among others (Singleton et al. 2004; Chevalier et al. 2023). In contrast, although some studies suggested a higher prevalence of cardiovascular autonomic failure in PD associated with heterozygous glucocerebrosidase (GBA) mutations (Petrucci et al. 2020; Maldotti Dalla Corte et al. 2025), other investigations employing detailed electrophysiological assessments have not confirmed this association (Giannini et al. 2024). Finally, PD linked to LRRK2 or PRKN (parkin) mutations has been consistently associated with a lower frequency of cardiovascular autonomic failure compared with matched sporadic PD cohorts (Del Sorbo et al. 2007; Tijero et al. 2013, 2015).

Left untreated, cardiovascular autonomic failure has a major impact on the quality of life, morbidity and mortality of affected PD individuals. It interferes with activities of daily living, gait and cognitive performance, increases the risk of syncope related falls, hypertensive emergencies, and contributes to the development of coat-hanger and low back pain (Poda et al. 2012; Fanciulli et al. 2013; Ricci et al. 2015; Hauser et al. 2018; Palma et al. 2020; Raccagni et al. 2020; Campese et al. 2024). In clinical practice, a high level of awareness and regular screening for symptoms suggestive of cardiovascular autonomic failure are therefore important to ensure both timely diagnoses, reduce the burden of autonomic symptoms, and positively influence the prognosis. Future studies will address current knowledge gaps in the non-pharmacological and pharmacological treatment of both OH and SH, and better appraise the influence of advanced, device-aided PD therapies on the severity of autonomic symptoms.

## Data Availability

No datasets were generated or analysed during the current study.
